# The impact of altering participant MRI scanning position on back muscle volume measurements

**DOI:** 10.1259/bjro.20210051

**Published:** 2022-03-30

**Authors:** Salman Alharthi, Jude Meakin, Chris Wright, Jonathan Fulford

**Affiliations:** ^1^ Medical Imaging Department, Medical School, University of Exeter, Exeter, United Kingdom; ^2^ Radiology and Medical Imaging Department, College of Applied Medical Sciences, Prince Sattam bin Abdulaziz University, Al-Kharj, Saudi Arabia; ^3^ Physics and Astronomy Department, University of Exeter, Exeter, United Kingdom; ^4^ NIHR Exeter Clinical Research Facility, University of Exeter, Exeter, United Kingdom

## Abstract

**Objectives:**

Muscle volume may reflect both strength and functional capability and hence is a parameter often measured to assess the effect of various interventions. The aim of the current study was to determine the sensitivity of muscle volume calculations on participant postural position and hence gauge possible errors that may arise in longitudinal studies, especially those where an intervention leads to large muscle changes and potentially the degree of spinal curvature.

**Methods:**

Twenty healthy participants (22–49 years, 10 male and 10 female), were recruited and MRI images acquired with them lying in four different positions; neutral spine (P1), decreased lordosis (P2), increased lordosis (P3) and neutral spine repeated (P4). Images were analysed in Simpleware ScanIP, and lumbar muscle volume and Cobb’s angle, as an indicator of spine curvature, determined.

**Results:**

After comparing volume determinations, no statistically significant differences were found for P1 - P2 and P1 - P4, whereas significant changes were determined for P2 - P3 and P1 - P3. P2 and P3 represent the two extremes of spinal curvature with a difference in Cobb’s angle of 17°. However, the mean difference between volume determinations was only 29 cm^3^. These results suggest the differences in muscle volume determinations are generally greater with increasing differences in curvature between measurements, but that overall the effects are small.

**Conclusions:**

Thus, generally, spinal muscle volume determinations are robust in terms of participant positioning.

**Advances in knowledge:**

Differences in muscle volume calculations appear to become larger the greater the difference in spinal curvature between positions. Thus, spinal curvature should not have a major impact on the results of spinal muscle volume determinations following interventions in longitudinal studies.

## Introduction

Although many muscles are connected to the spine, research studies typically focus on the erector spinae, multifidus and psoas. The erector muscles (spinalis, longissimus and iliocostalis) and multifidus muscles are both located laterally to the lumbar spine and are responsible for stabilising the spine. The psoas muscle is one of the flexor muscles and arises from the lumbar spine. It helps to flex the trunk and provides stability and balance to the whole body when in a relaxed upright standing position.^1–4^

The size of muscles typically reflects their strength and functional capability. Thus, studies that have assessed the impact of disease, exercise or immobilisation on spinal muscle function have generally measured muscle size as a direct indicator.^3,5–7^ Typically, this is achieved via the measurement of muscle cross-sectional area images obtained from computed tomography (CT), ultrasound or magnetic resonance imaging (MRI).

Within the literature, there are a large number of studies examining the magnitude of muscle size modifications following a range of interventions that involve obtaining data at a number of time points.^6,8–13^ All of these studies however, rely on the assumption that the effect of intervention is much greater than any errors that may arise from differences that occur because of variations between visits in participant position when being scanned. These difference may arise, for example, from differences in spinal curvature that result from modifications in spinal muscle size, as may be associated with an exercise intervention.

The current study therefore aims to investigate the sensitivity of lumbar muscle volume measurements on the participant position during scanning, and the subsequent impact this might have on the ability to detect volume changes within longitudinal studies. To place the results into context, muscle volume differences arising from varying participant positions will be compared with intrinsic quantification variability by undertaking multiple repetitions of the volume analysis on a subset of the acquired data sets.

## Methods and materials

### Data

Twenty healthy participants were recruited (10 male and 10 female), without any history of back pain, ranging in age from 22 to 49 years old (mean ± sd 38 ± 8 years). The study was approved by the institutional ethics committee, and all participants gave informed consent.

### Data acquisition

Participants were invited to have MR scans of their lumbar spine using a 1.5T research scanner (Intera, Philips, The Netherlands). Once participants had been positioned within the scanner, survey scans were acquired to allow appropriate positioning of the subsequent anatomical scans. Scans were acquired, centred at L3/L4, using a spine coil consisting of a 5-element surface coil array. A turbo spin-echo sequence was run to provide images in the sagittal plane in order to have clear disc visualisation, thereby allowing the angle between discs to be determined (11 slices, repetition time (TR) = 400 ms and echo time (TE) = 8 ms, 4-mm slice thickness, 0.4 mm slice gap). Subsequently, a 3D T1 gradient echo (GRE) sequence was acquired in the axial plane for muscle volume measurements (TR = 20 ms, TE = 4.5 ms, voxel size 0.5 mm, with 59 contiguous 5 mm slices).

### Positioning protocol

The scanning protocol was repeated four times with the participant lying supine on the scanner bed in different positions; P1: neutral spine, P2: decreased lordosis, P3: increased lordosis and P4: neutral spine repeated. A total scanning duration of approximately 7 min was required for each position.

Initially, images were acquired with the participant in a flat, supine position (P1). In the second position (P2) participants were asked to lie in a supine position and then hug their knees to their chest. Wedges were pushed towards their pelvis to support its posterior tilt. Subsequently, they were asked to uncurl their legs and lie down with instructions to try and push their lower back towards the bed, with an additional wedge placed beneath the knees. For the third position (P3), participants were asked to sit upright with their legs extended in front, and wedges were pushed towards their pelvis from behind to support its anterior tilt. They were then asked to lie down with instructions to try and hold an exaggerated lumbar curve. Finally, the initial neutral position with no wedges was repeated (P4).

### Data analysis

**Figure 1.**
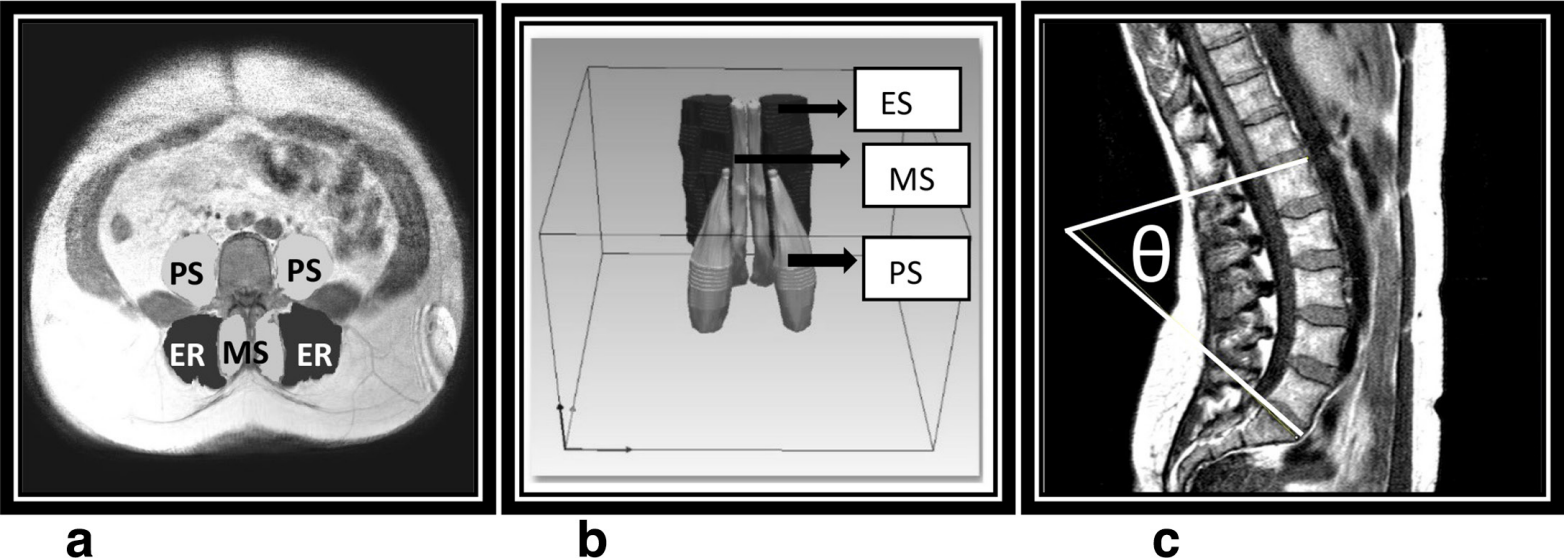
Axial cross-sectional image, illustrating erector spinae (ER), multifidus (MS) and psoas muscles (PS) in (**A**), a 3D Image in (**B**) and lumbar lordosis angle measurement in (**C**).

Following data collection, the images were transferred to analysis software Simpleware ScanIP (O-2018.12, Synopsys, Inc.) in order to measure the lumbar spine angles and the spinal muscle volume. Each image set was individually imported as a DICOM file. Separate masks were then utilised for each muscle type: erector spinae (ER), multifidus (MS) and psoas (PS). The paint tool was used to manually paint the muscles within the axial slices as shown in Figure 1. However, some of the muscle edges were blurred, making it challenging to quantify the boundaries. Subsequently the interpolation toolbox within ScanIP was used to reduce the time required for segmentation of each slice as well as improving the accuracy in areas where there was no clear muscle boundary. Hence, the process undertaken involved the researcher initially manually painting every second slice to ensure all muscle boundaries were defined, flood filling the areas, and then utilising the Interpolation toolbox to define muscles areas in the slices in which boundary definition had not been undertaken. Although this is reported to reduce the time taken compared with manually painting all slices while maintaining high quality segmentation Synopsys (2018), each scan still took 75 to 120 min to segment.

Angulation measurements were undertaken to examine the differences in spinal curvature between the participant positions. Measurements were undertaken on the sagittal images, with two lines being drawn, one tangential to the superior endplate of L1 and one tangential to the superior endplate of S1, and their angle of interception, lumbar lordosis, calculated, as shown in Figure (1 C).

### Analysis repeatability

Sixteen out of the 80 data sets were selected randomly. These data sets were then segmented twice to investigate the repeatability of the analysis approach when determining muscle volume. All repeated measurements were done using the same procedure described above at different times separated by at least one week.

## Statistics

All statistical tests were undertaken using SPSS (version 26, IBM). Total muscle volume was calculated by summing the individual muscle measurements for each participant (erector spinae, multifidus and psoas). Means and standard deviations for total volume and spinal curvature were calculated for each participant position and paired t-tests utilised to investigate whether was there any differences between pairs of positions, with significance defined as *p* < 0.05.

Bland-Altman plots were obtained to examine the level of agreement between repeated measurements.^14^ Thus, this was utilised to assess the variation between volume measurements for positions P1 and P4 within the positioning aspect of the protocol and for the assessment examining repeated analysis of the same data sets. This included the calculation of 95% confidence limits and interclass correlation coefficients (ICC). In order to provide an indication of the measurement errors associated with the positioning and analysis aspects three further parameters were determined. Typical error of measurement (TEM) was calculated as the standard deviation of the differences between repeated measures divided by √2.^15^ The standard error of measurement (SEM) calculated using: SEM= ((Σ (difference between measurement pairs)^2)/2 x number of participants))^1/2^.^16^ The smallest change that could be detected (SDC) from a single measurement was calculated from SDC=2^1/2^ x t_(1-α/2)_ x SEM where t_(1-α/2)_ represents the 95% confidence value for the t distribution.^16,17^ For a sample size of 20, and thus 19 degrees of freedom, as was the case for the positioning aspect of the study this has a value of 2.09. For the analysis repeatability, the sample size was 16, leading to 15 degrees of freedom and hence a value of 2.13. The value of SDC when looking for differences between groups was determined by the individual measurement of SDC/√n where n represents the number of participants.^14,16–18^

## Results

### Participant position

**Figure 2.**
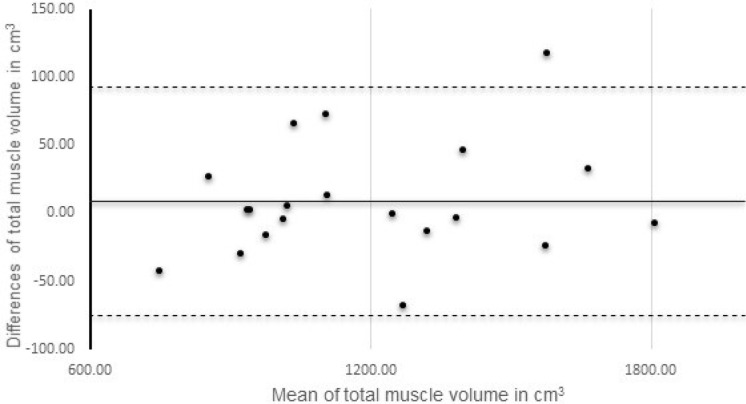
Bland Altman plot, comparing repeat measurements of spinal muscle volumes with participants lying in the same position. The straight line corresponds to the mean difference between the measurements, while dotted lines corresponding to the 95% limits of agreement ( ± 1.96 standard deviations).

The results from the muscle volume determinations for the 20 participants are given in Tables 1 and 2 displaying averages for each position and the differences for values between positions. As anticipated, changing participant position leads to modifications in spinal curvature, as shown in Tables 1 and 2, with significant differences between positions P1 and P2, P1 and P3 and P2 and P3, but not between the repeated positions P1 and P4. As shown in Table 2, these curvature alterations lead to significant differences in muscle volumes between P1 and P3 and P2 and P3, whereas no differences were determined between P1 and P2 and P1 and P4. For positions P1 and P4, the Bland-Altman plot is shown in Figure 2, illustrating a high level of agreement, with mean differences of 8.67 cm^3^ and 95% coefficient intervals of 92.21 and −74.86 cm^3^. SEM was determined as 30.01 cm^3^, TEM 30.14 cm^3^, and SDC was 89.95 cm^3^ for a single measurement and 20.11 cm^3^ for a group of 20.

**Table 1.** Muscle volume and lumbar lordosis measurements. Neutral spine position - P1, decreased spine lordosis position - P2, increased spine lordosis position - P3 and repeat of neutral spine position - P4

Participant position comparison	Muscle volume		Spinal curvature	
	Differences: mean ± sd in cm^3^	t-statistic (P)	Differences: mean ± sd in degrees	t-statistic (P)
P1 - P2	−9.9 ± 3.8	−1.74 (0.097)	8.7 ± 0.2	8.34 (0.001)
P1 - P3	18.9 ± 2.1	2.13 (0.046)	−8.3 ± 1.3	−9.16 (0.001)
P1 - P4	8.6 ± 8.9	0.91 (0.374)	1.4 ± 0.2	1.92 (0.076)
P2 - P3	28.8 ± 25.5	3.04 (0.006)	−17.0 ± 1.1	−13.98 (0.001)

**Table 2.** Mean and standard deviation (sd) differences in muscle volume and spinal curvature for different participant positions. The t-statistics and *P* values result from paired t-tests with *n* = 20. P1: Neutral spine position, P2: decreased spine lordosis position, P3: increased spine lordosis position, and P4: repeat of neutral spine position

Participant position	Volume: mean ± sd in cm^3^	Spinal curvature: mean ± sd in degrees
P1	1200 ± 300	54 ± 11°
P2	1210 ± 304	45 ± 10°
P3	1181 ± 278	62 ± 8°
P4	1191 ± 291	52 ± 11°

### Repeatability

**Figure 3.**
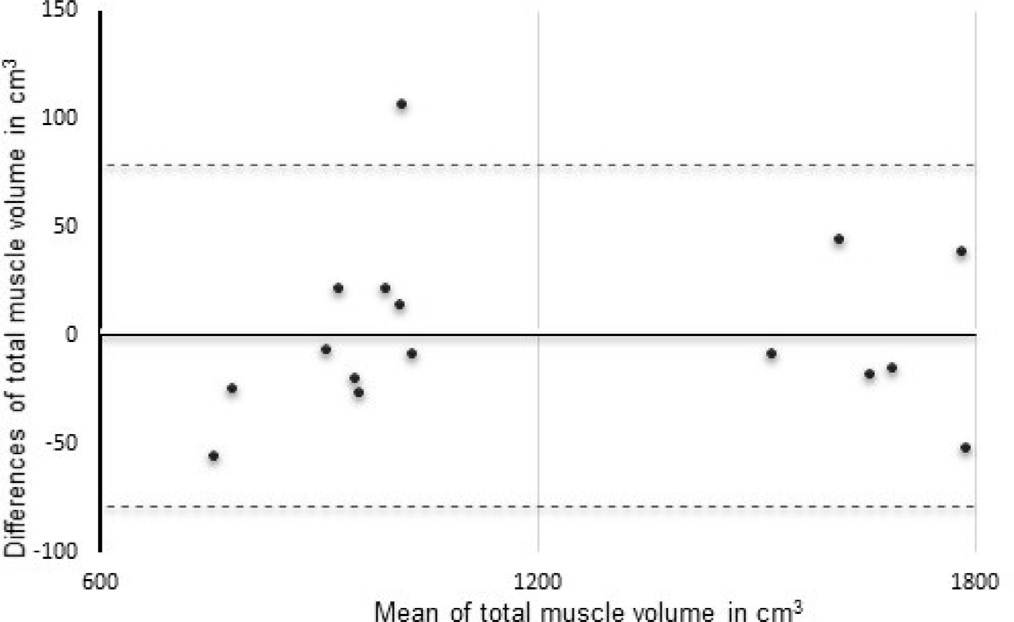
Bland Altman plot, comparing repeat analysis of total muscle volumes from single data sets measurements. The straight line corresponds to the mean difference between the measurements, while dotted lines corresponding to the 95% limits of agreement ( ± 1.96 standard deviations).

The first set of muscle volume determinations resulted in a value of 1212 ± 383 cm^3^ (mean ± sd) with the repeat measurement giving a value of 1211 ± 382 cm^3^, thus displayed only a small variation of 0.1 %, with a high correlation with ICC 0.99. The Bland Altman plot illustrated in Figure 3 revealed a high level of agreement between the repeated measurements. The mean difference was 0.11 cm^3^ with 95% limits of agreement between −78.70 and 78.91 cm^3^. SEM was 27.52 cm^3^, TEM was 28.43 cm^3^, while the SDC for a single measurement was 82.53 cm^3^ and 18.45 cm^3^ for a group of 20.

### Discussion

This study has examined the effect of participant position on lumbar muscle volume measurements. The main finding is that although differences in muscle volume appear to become larger the greater the difference in spinal curvature, overall, the participant position has a relatively small effect on muscle volume. When altering the position from neutral to decreased lordosis a small, significant increase in volume was measured of 19 cm^3^. In comparison, positions P2 and P3 which represent the two extremes of participant position, with exaggerated decreased and increased curvature, resulting in 17 degrees difference between the two, still only resulted in a mean difference of 29 cm^3^.

The main aim of the study was to compare volume changes resulting from positioning changes to volume changes typically seen in interventional longitudinal studies. The 29 cm^3^ difference seen between the extreme curvature positions, which represent a degree of curvature difference much greater than would be anticipated between longitudinal measurements, corresponds to approximately 2% of the mean total muscle volume of 1200 cm^3^. In comparison, differences in muscle volume typically reported in the literature for intervention studies tend to be much larger than this. For instance, Kim et al.^11^ reported increases of approximately 6.5 and 4% in multifidus and psoas respectively after two months of spinal stabilisation exercise and Danneels et al.^8^ , reported a 6.6% increase in the multifidus following 10 weeks of stabilisation training combined with dynamic-static resistance. When assessing multifidus changes following different exercise regimes, Chung et al.^10^ reported a significant increase at the level of L4 of 18% following dynamic stabilisation exercises and 8.2% with more passive stabilisation exercise, while at the L5 level it increased by 23% and 8.7% for the respective groups. When examining the impact of inactivity, Cao et al.^19^ determined that after 28 days bed rest a 7.7% decrease in erector spinae CSA was seen compared with a 6.8% decrease when bed rest was combined with head-down-tilting exercise.^19^ Likewise, in 2016 Holt et al, reported, that following 2 months of bed rest, a 10.9±3.4% decrease in erector spinae muscle was seen, compared with a 4.3±3.4% decrease when 4 days of bed rest was combined with 3 days of aerobic and resistive training every week.^20^

In terms of the reliability of the measurements, both repeating the acquisition of data and repeating the analysis procedure on single data sets resulted in an SDC of 80–90 cm^3^ for a single measurement, corresponding to a value of approximately 20 cm^3^ when comparing groups of 20 participants. Hence, in order for future intervention studies to be able to detect a significant impact, effects greater than these thresholds should be aimed for.

Overall the study aimed to examine the impact of posture on back muscle volume determinations when undertaking longitudinal measurements. The findings suggest that in general, longitudinal measurements should be comparable, except potentially under cases of extreme variations in spinal curvature. Hence, for example, if a participant is having back surgery, and only requires back support on their pre visit to alleviate pain resulting in different degrees of spinal curvature between visits, there may be significant, but small changes in muscle size determination.

## Conclusion

Differences in muscle volume calculations appear to become larger the greater the difference in spinal curvature between positions. However, the effect is relatively small in terms of absolute muscle volume differences. Generally therefore, spinal muscle volume determinations are robust in terms of their sensitivity to participant position and subsequent curvature and should not have a major impact on the results of intervention studies.
